# Enhancing condom use experiences among young men to improve correct and consistent condom use: feasibility of a home-based intervention strategy (HIS-UK)

**DOI:** 10.1186/s40814-018-0257-9

**Published:** 2018-03-07

**Authors:** Nicole Stone, Cynthia Graham, Sydney Anstee, Katherine Brown, Katie Newby, Roger Ingham

**Affiliations:** 1 Centre for Sexual Health Research, Department of Psychology, University of Southampton, Southampton, UK; 2 Centre for Advances in Behavioural Science, Coventry University, Coventry, UK

**Keywords:** Condom, Condom use, Fit and feel, Intervention, Sexual health, Sexually transmitted infections, Behaviour change, Health promotion, Young men, Feasibility

## Abstract

**Background:**

Condoms remain the main protection against sexually transmitted infections (STIs) when used correctly and consistently. Yet, there are many reported barriers to their use such as negative attitudes, reduced sexual pleasure, fit-and-feel problems and erection difficulties. The UK home-based intervention strategy (HIS-UK) is a behaviour change condom promotion intervention for use among young men (aged 16–25 years) designed to increase condom use by enhancing enjoyment of condom-protected intercourse. The objective of this feasibility study was to test HIS-UK for viability, operability and acceptability. Along with an assessment of the recruitment strategy and adherence to the intervention protocol, the study tested the reliability and suitability of a series of behavioural and condom use outcome measures to assess condom use attitudes, motivations, self-efficacy, use experience, errors and problems and fit and feel.

**Methods:**

The HIS-UK intervention and associated assessment instruments were tested for feasibility using a single-arm, repeated measures design with baseline measurement and two follow-up measurements over 3 months. A 3-month target of 50 young men completing the baseline questionnaire was set. Twenty process and acceptability evaluation interviews with participants and health promotion professionals were conducted post trial.

**Results:**

Of the 61 young men who registered for the study, 57 completed the baseline questionnaire and 33 met with the study researcher to receive the HIS-UK condom kit. Twenty-one young men remained for the duration of the study (64% retention). The Cronbach’s alpha scores for the condom use outcome measures were 0.84 attitudes, 0.78 self-efficacy, 0.83 use experience, 0.69 errors and problems and 0.75 fit and feel. Participant and health professional feedback indicated strong acceptability of the intervention.

**Conclusions:**

The feasibility study demonstrated that our recruitment strategy was appropriate and the target sample size was achieved. Adherence was favourable when compared to other similar studies. The condom use measures tested proved to be fit-for-purpose with good internal consistency. Some further development and subsequent piloting of HIS-UK is required prior to a full randomised controlled trial, including the feasibility of collecting STI biomarkers, and assessment of participant acceptance of randomisation.

**Trial registration:**

Research registry, RR2315, 27th March 2017 (retrospectively registered).

## Background

The Framework for Sexual Health Improvement in England recognises human immunodeficiency virus (HIV) and sexually transmitted infections (STIs) as important public health concerns, especially among young people [1]. Despite a large reduction in the rates of teenage pregnancies in the UK, STI rates remain high. According to a recent Public Health England report, there were approximately 420,000 STI diagnoses made in England during 2016 and the impact of STIs remains greatest among young heterosexuals under the age of 25 years and in men who have sex with men. Compared to people aged 25–29 years, current rates of STI diagnoses among 16- to 24-year-olds are twice as high in men and seven times higher in women [2].

Male condoms can be highly protective against the transmission of STIs when used correctly and consistently [3]. However, there is substantial evidence that condoms are often not used properly and there are many barriers to their use [4]. Research has linked problems with condom ‘fit and feel,’ erection difficulties, negative attitudes, and reduced sensation and sexual pleasure with inconsistent and non-condom use [4]. The effectiveness of condoms is also reduced by incomplete use (not using condoms from start to finish of penetrative vaginal or anal intercourse), which is more commonly described by men who report negative experiences and problems when using condoms [4, 5].

One of the Department of Health’s current priorities is to reduce STI rates using evidence-based preventative interventions [6]. Current national guidelines regarding behavioural interventions to prevent STIs are, however, limited. Historically, many health promotion interventions have tried to improve knowledge and skills to promote effective condom use; systematic reviews of the efficacy of these interventions have produced mixed results [7–9]. Poor quality trials and a failure to identify the active components (behaviour change techniques) of existing interventions have reduced the ability of previous work to inform future intervention development. Moreover, there has been a tendency to focus on resource-intensive interventions; in practice, health promotion staff may not have the time or the resources to fully engage with ‘high-demand’ intervention programmes. Yet there is emerging evidence that brief interventions designed with identifiable and evidence-based components can reduce STI acquisition among young people [10–12].

Recent National Institute of Health and Care Excellence (NICE) guidelines on condom distribution stress the need to teach young people to use condoms effectively and safely (using education, information and demonstrations), and to provide a range of condoms and lubricants to meet needs [13]. NICE also recommends that one-to-one condom promotion discussions focusing on skills acquisition, communication skills and motivation to adopt safer sexual behaviours should be provided as part of routine care of those at elevated risk of STIs [13, 14].

In light of the reported obstacles to correct and consistent condom use [4], there is a need to develop brief interventions that focus on condom barriers as important determinants of condom use behaviour. The Kinsey Institute Homework Intervention Strategy® (KIHIS) is a brief behaviour change condom promotion intervention, developed in the US to improve condom skills, enjoyment and self-efficacy among young men [15, 16]. KIHIS is novel in that it aims to increase condom use by enhancing the fit and feel of condoms and, thereby, outcome expectancies related to enjoyment of sex whilst using condoms [17, 18]. The primary philosophy behind the KIHIS intervention is to place the impetus for behaviour change on individuals by encouraging solitary practice and experimentation with condoms. The model adopted is taken from the behavioural therapy approach most commonly used to treat sexual problems and which incorporates homework assignments (or ‘directed practice’) [19]. In common with this approach, the KIHIS includes behavioural exercises designed to promote condom experimentation and increase an individual’s focus on pleasurable sensations whilst using condoms. The KIHIS programme has three elements: self-practice with condoms in ‘no pressure’ situations, experimentation with different condom and lubricant brands (to address issues of fit and feel) and encouragement to focus on the physical sensations whilst using condoms.

KIHIS has demonstrated early evidence of efficacy in studies conducted in the US and Canada [15, 16]. A small pilot study involving young heterosexual men found that the intervention led to significant improvements in condom use experiences, self-efficacy and condom fit and feel, as well as a reduction in breakage and erection problems [15]. A study of young men who have sex with men demonstrated that KIHIS increased condom use and improved attitudes toward condoms and condom use self-efficacy [16].

The objective of the HIS-UK study was to adapt and manualise the KIHIS into a cultural- and health care appropriate intervention for delivery to young men (aged 16–25 years) within community settings in the UK and test it for viability, operability and acceptability [20, 21]. The study involved a series of work packages. Firstly, we conducted a systematic review to synthesise available literature on the effects of condom-focused, pragmatic behavioural interventions of relevance to the UK context (Anstee S, Graham CA, Stone N, Shepard J, Brown K, Newby K, Ingham R. The evidence for behavioural interventions addressing condom use fit and feel issues to improve condom use: A systematic review. Submitted). This was accompanied by a behaviour change taxonomy coding exercise to identify active components of effective interventions that could be incorporated into HIS-UK [22]. Secondly, a user consultation and design phase, involving discussions and workshops with young men, youth workers, and health promotion professionals, was carried out to ensure the HIS-UK intervention and the associated research study was developed in genuine partnership with those for whom it was intended. The aim of the third, feasibility, work package was to test the study recruitment and retention strategy, intervention delivery protocol, participant adherence, data collection and assessment tools, proposed outcome measures and analysis methodology. The final work package was a post-intervention qualitative process evaluation to assess the acceptability of HIS-UK implementation, involving in-depth interviews with study participants and health promotion professionals. Findings from the feasibility study, lessons learned from the process evaluation interviews and conclusions drawn about the suitability of taking HIS-UK to a full trial are presented here. Outcomes from the systematic review and a full description of the development of the intervention, including the behaviour change techniques incorporated, will be reported elsewhere.

## Methods

### Sample recruitment

Recruitment was focused in and around two UK cities, Southampton and Coventry. To determine feasibility of recruitment, we tested whether our advertising and recruitment strategy could attract a minimum of 50 eligible young men to register their interest in the study and complete the baseline questionnaire within a 3-month period. Furthermore, a sample size > = 20 would ensure that the reliability estimates of our outcome measures would be representative of a main study sample [23]. Posters, advertisements and business cards were placed in community and educational settings attended by young men within the two localities, and study staff attended local educational and community events to raise awareness and promote recruitment. The study had its own information and recruitment website which was advertised via relevant Facebook pages, on Twitter and through the social media of local community and educational agencies. Interested young men who were aged 16–25 years, sexually active (had engaged in vaginal and/or anal intercourse), who sometimes had sex without condoms and who did not report an allergy to any type of lubricant or condom (latex or non-latex) were eligible to register their interest in participating. Approval for the study was obtained from the University of Southampton Ethics Committee (ID: 17504).

### Intervention delivery and follow-up

A single-arm, repeated measures study design with baseline outcome measurement and two follow-up outcome assessments was employed. Young men meeting the study eligibility criteria, giving informed consent and registering their interest were prompted to complete an online baseline questionnaire (T1). This asked for basic demographic information, including age, ethnicity, employment status and educational attainment. They were also asked a series of questions about their sexual history, including sexual orientation, number of lifetime sexual partners (defined as someone with whom they had experienced penetrative anal or vaginal intercourse), lifetime experiences of pregnancy and STIs, sexual education received and past use of condoms and lubricants. Participants’ current relationship status, recent (in the last 4 weeks) sexual activity, STI testing and use/non-use of contraception (including condoms) were also assessed at T1, along with their motivation for taking part in the research (open text box response).

At baseline, participants were additionally asked about their condom use attitudes, self-efficacy, condom use intentions and motivation and details of any sexual activity and condom use (including experiences, errors and problems) that had occurred in the last four-week period. The outcome measures selected for testing were taken from previously validated assessment instruments, including the Multidimensional Condom Use Attitudes Scale, the Condom Use Self-Efficacy Scale, the Condom Barriers Scale, the Condom Use Errors and Problems Survey and the Condom Fit and Feel Scale [15, 16, 19, 24–30]. *Lifeguide* software was used for the purposes of administering the online questionnaires [31].

All participants who successfully completed T1 and who provided valid contact details (e-mail and/or mobile number) were contacted by the study research assistant (RA) to arrange a suitable time and location to meet. During a brief face-to-face meeting with the RA (approximately 20 min) participants received an introduction to HIS-UK, a condom kit containing eight different types of condoms and three different types of lubricants and a condom information/instruction leaflet. To ensure condom competency among all participants, each received a condom application demonstration by the RA using a penile demonstrator, which they were asked to repeat back to the RA until no errors were made. They were also given verbal (as well as written) instructions for what to do during the subsequent 2-week condom testing and rating period: to practice applying, using (masturbating with) and removing each of the condoms provided in the condom kit in ‘low pressure’ situations (i.e. not in the presence of a sexual partner); to try out the different lubricants; and to complete online rating forms within 24 h of each condom test. Each participant was asked to try out all the different condom types during the 2-week testing period and submit ratings for each. *Lifeguide* facilitated automated texts and e-mails to prompt participants to complete the required tests and ratings. Protocol compliance was defined as a minimum of three submitted rating forms and full compliance as the submission of eight.

The condom testing exercises were designed to enable participants to (1) discover the right/best condom for fit and feel, (2) experiment with different lubricants, (3) test techniques of application and removal and (4) focus on pleasurable sensations and reflect upon how condoms and lubricants could be used for the benefit of both their partners, and their own, enjoyment and thereby challenge beliefs that condoms ‘spoil’ sex.

The condom rating survey began by asking for some simple details about each test: the condom type, how long the test lasted and whether or not a full test was completed (i.e. whether the condom was applied to the penis). Participants were then presented with a series of 17 statements about the condom and asked how strongly they agreed or disagreed with each, using a 5-point scale. The statements were designed to encourage participants to think carefully about how the condom felt, how it fitted and how easy and enjoyable it was to use. Example statements included (i)* this condom was easy to put on*, (ii)* this condom was comfortable*, (iii)* this condom was too long/short/tight/loose*, (iv) *this condom hurt my penis*, (v)* this condom had an unpleasant smell*, (vi)* I stayed aroused whilst putting this condom on *and (vii) *this condom decreased sensation*. Participants were then asked if they had used a lubricant with the condom. If they had, they were prompted with a series of questions about usage, including (i) where the lubricant was applied, (ii) how pleasant was the odour, (iii) did it have a good texture, (iv) did it last/not dry up and (v) did it enhance pleasure.

At 4 weeks post-rating, and then again at 8 weeks post-rating, participants received automated texts and e-mails prompting them to complete a follow-up assessment questionnaire (T2 and T3 respectively) in which they were once again asked to complete the series of behavioural, condom use experience, attitudinal, intentions and self-efficacy outcome measures (as per T1), along with details of any partnered sexual activity and condom use that had occurred in the previous 4-week period.

Previous longitudinal studies of sexual health and condom promotion have struggled to achieve high participant retention rates (for example, a 50% retention rate was achieved by Bailey et al. at 12-month follow-up) [32, 33]. What is more, it has been well established that selected groups, in particular young people and young men, are more likely to drop out of studies [34]. As such, the study was incentivised and a minimum target retention at T3 of two thirds (67%) was set. Participants who successfully completed T2 were sent a condom selection gift pack as a thank you for their participation. Those who went on to complete the final T3 questionnaires were sent a £20 gift voucher.

### Process evaluation

Following completion of the data collection activities, young men who participated in the study and health promotion professionals involved in recruitment were invited to participate in a follow-up process evaluation interview. The purpose of the interview was to assess satisfaction and acceptability of the intervention, the study methodology and the research design. Fifteen participants were interviewed over the telephone, and five health professionals were interviewed face to face. Each interview lasted between 30 and 40 min and was digitally recorded with consent. All participating young men received a £10 voucher. The interview schedule was designed using the evaluation framework proposed by Saunders et al. [35], providing useful components with which to measure success and to identify where adjustments should be considered in future testing of the intervention.

### Outcome measures

The primary objective of HIS-UK is to improve the use of condoms during episodes of vaginal or anal intercourse, and two standard condom use outcome measures were trialled during the feasibility. Consistency of use was determined by the responses given to the following questions: *How many times have you had sex (anal or vaginal intercourse) in the last 4 weeks* and *Did you use a condom during sex in the last 4 weeks? If yes, how many times did you use a condom?* The secondary objectives, through which the primary objective is likely to be realised, reflect condom competency, satisfaction and enjoyment. These were measured using a single question regarding lubricant use and a series of condom use scale items assessing negative condom use attitudes, motivations, self-efficacy, negative use experience, errors and problems and poor fit and feel. Similar scale items have been used in previous KIHIS studies [18, 19].

#### Condom use experience scale

Condom use experience was assessed using a seven-item subscale of the validated Condom Barriers Scale [24, 25], measured on a 5-point Likert scale (1—strongly disagree, 5—strongly agree). Example items include *Condoms don’t fit properly*, *Condoms rub and cause irritation* and *Condoms interrupt the mood*. A mean score for the seven individual items was created, with a higher score indicating more negative use experience. The mean score from the T1 sample was 3.34 (range 2.00–4.86). The Cronbach’s alpha was 0.83, 95% CI [0.76–0.89], indicating a high level of internal consistency for the scale with the T1 sample.

#### Condom use errors and problems scale

Fourteen items from the condom use errors and problems survey [26, 27] were used. Example items included *Application of the condom the wrong way up*, *Losing an erection whilst applying a condom*, *Using a condom from the start to finish of intercourse* and *Breakage of the condom during intercourse*. Participants were asked to report (using a binary yes/no response) whether any of the statement items had occurred or been experienced during last condom use. Responses from nine of the statements were combined to create the final errors and problems scale to achieve the greatest level of internal consistency (M = 3.13, range 0–8, Cronbach’s alpha 0.69, 95% CI [0.55–0.80]). A higher score on the measure reflected more reported errors and problems.

#### Condom use fit-and-feel scale

Six statement items from the Condom Fit and Feel Scale, exploring men’s experiences with the fit and feel of condoms used during the previous 4-week period, were tested for suitability [28]. Responses were provided on a frequency 4-point Likert scale (1—never applies to me, 4—always applies to me). Example statement items included *Condoms are too short for my penis* and *Condoms are too tight for my penis*. The resulting scale which proved to be the most robust measure of fit and feel included four of the six items (M = 8.98, range 4–16, Cronbach’s alpha 0.75, 95% CI [0.62–0.84]). Statements relating to condoms being *too long* and *too loose* were removed. A high score on the scale reflected poor fit-and-feel outcomes.

#### Condom use attitude scale

The Condom use attitudes scale was originally adapted from the Multidimensional Condom Attitude Scale [19] and designed as a seven-item scale to measure negative attitudes toward condoms. In an earlier KIHIS study, Emetu et al., however, used a shorter five-item version [16]. Both versions were tested and the five-item version provided a good fit to our data, with seven Likert scale response options (1—strongly agree, 7—strongly disagree). Example items included *Condoms ruin intercourse*, *Condoms make sex less stimulating* and *Condoms interrupt the mood*. A higher score indicated more negative attitudes (M = 4.58, range 2–7). The Cronbach’s alpha was 0.84, 95% CI [0.76–0.90], indicating a high level of internal consistency for the scale with the T1 sample.

#### Condom use self-efficacy scale

Condom use self-efficacy was assessed by an established seven-item measure of self-efficacy designed to assess ability to apply condoms correctly [29, 30]. Items asked participants how difficult or easy (1—very difficult, 5—very easy) they would find certain actions, for example, *Find condoms that fit properly*, *Apply condoms correctly* and *Keep an erection when using a condom*. A mean score for the seven individual items was created, with a higher score indicating greater self-efficacy. The mean score from the T1 sample was 3.65 (range 1.57–5.00). Cronbach’s alpha for the T1 sample was 0.78, 95% CI [0.67–0.86].

#### Condom use motivation scale

This was a single question asking young men to report, using a five-point Likert scale (1—strongly disagree, 5—strongly agree), how motivated they were to use condoms correctly, and how motivated they believed their partners were to use condoms correctly. Higher scores indicated greater reported motivation to use condoms.

### Data analysis

The feasibility study was designed to test our procedures and data collection methodology and to determine whether our feasibility targets had been met. Responses to the questionnaires and condom rating forms were collected using *Lifeguide* software and imported into the IBM Statistical Package for the Social Sciences v20 for analysis.

Differences in the demographics (age, sexual orientation, employment and education), sexual history and behaviour (pregnancy and STI diagnoses, sexual partnerships, episodes of intercourse, use of contraception, condoms and lubricant) and the condom use scale items were compared at baseline (T1) between young men who registered and received HIS-UK and those who registered but did not receive the intervention, using unpaired *t* tests and Fisher’s exact tests. Similar group comparisons were made between participants based on the degree of intervention adherence during condom testing and again at T2.

The aim of the feasibility study was not to evaluate the efficacy of HIS-UK to change condom use behaviour, attitudes, use experience or self-efficacy—indeed that is the purpose of a planned larger randomised controlled trial. However, to assist in our assessment of suitability of the proposed outcome measures and methods of data analysis and to identify potential questionnaire omissions, we ran a series of simple comparative analyses on the T1, T2 and T3 data using paired *T* tests and generalised linear modelling and generalised estimating equation procedures, to allow for the analysis of repeated measurements over time. The scale items measured using Likert scale responses were compared over three time points using repeated measures ANOVA; all other response types were examined using a generalised estimating equation with repeated measures and time as the design factor (binary data: binomial as the distribution and logit as the link function; ordinal outcomes: multinomial as the distribution and cumulative logit as the link function). Due to the preliminary nature of these analyses and the small sample sizes involved, no additional co-variates were controlled for.

The qualitative process evaluation interviews with participants and health promotion professional were semi-transcribed and coded for key content and analysis under the following themes proposed by Saunders et al. [35]: context, fidelity, recruitment, exposure, satisfaction, reach and content.

## Results

### Participant recruitment and retention

**Fig. 1:**
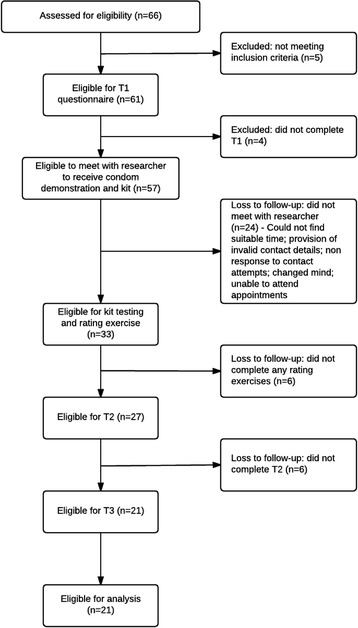
Summary of participant flow

During the 3-month recruitment period, 61 eligible young men registered to take part in the feasibility study and 57 (93%) went on to complete the T1 baseline questionnaire (see Fig. 1). Participants had a mean age of 19.4 years (SD = 2.2), 91% were White British and 84% self-identified as heterosexual. Participants had a median of three lifetime sexual partners. Two participants had previously been diagnosed with an STI, and seven were known to have had a partner become pregnant. All participants had used a condom on at least one occasion previously.

At the time of registration, 41 (72%) participants were currently in a relationship, of whom seven also reported having intercourse with other sexual partners. Nine participants (16%) had not had intercourse during the previous 4 weeks. Among the 48 who had, 24 (50%) had used a condom at the last intercourse.

Using an open text box response box (multiple reasons permitted), participants were asked about their motivation for involvement in the study. Just over half (*n* = 30) mentioned they were motivated by the free condoms, 21 cited the financial incentive offered, 18 because they wanted to ‘do good’ and ‘help out’ with the research, 15 mentioned the opportunity to try out new condom types, ten reported they wanted to become better users of condoms and two were motivated by the supply of free lubricant.

Of the 57 young men who completed T1, 33 (58%) met with the researcher and received the HIS-UK intervention and condom kit. The main reasons for not receiving HIS-UK included providing invalid contact details or not responding to researcher e-mails and telephone messages (*n* = 10), participants changing their mind and withdrawing from the study (*n* = 8) and missing pre-arranged appointments or not being able to find a suitable time to meet with the researcher (*n* = 7). During comparative analyses, only two significant differences were found between the men who received HIS-UK and those who did not. Receivers of HIS-UK were significantly more likely to live in areas of higher deprivation than non-receivers (mean = 6.62, SD = 2.41 vs M = 5.09, SD = 2.58: *t*(49) = 2.18; *p* = 0.034, 95% CI [0.11–2.94]) and more likely to report that their partners were highly motivated to use condoms correctly (M = 4.09, SD = 0.91 vs M = 3.48, SD = 1.20: *t*(54) = 2.17; *p* = 0.035, 95% CI [0.05–1.18]).

### Condom rating adherence

Of the 33 young men who received the condom kit, 27 (82%) completed at least one condom rating form and 23 (70%) completed three. Eighteen participants were fully compliant, testing all eight types of condoms provided during the 2-week condom testing period (55% adherence).

Young men who completed eight rating forms were compared to those who did not achieve the desired completion rate. Participants who were adherent reported significantly higher condom self-efficacy scores (i.e. greater ability) than those who were non-adherent (M = 3.97, SD = 0.69 vs M = 3.25, SD = 0.94: *t*(29) = 2.46; *p* = 0.020, 95% CI [0.12–1.32]), and reported significantly fewer condom use errors and problems (M = 2.12, SD = 1.69 vs M = 4.07, SD = 2.28: t(30) = 2.77; *p* = 0.010, 95% CI [0.51–3.39]).

A total of 197 condoms were rated by the intervention participants. Ninety-five percent were complete tests, i.e. the condom was successfully applied to the penis, and 85% of tests were performed alone, in the absence of a sexual partner. Incomplete tests were reportedly due to ill-fitting condoms (typically too small in size) and condom breakage on application. During 38 condom tests (19% of all tests), an additional lubricant was also tested; in 71% of these, test participants reported that the lubricant increased/enhanced pleasure.

### Follow-up questionnaire adherence (T2 and T3)

Twenty-one of the 33 men who received the condom kit completed T2 (64% adherence), all of whom went on to successfully complete T3. Comparative analyses between the participants who completed T2/T3 and those who did not showed no significant demographic or background differences. However, young men who completed the study were significantly more likely to adhere to the condom rating protocol and submit eight rating forms; 86% adherence as compared to 0% among young men who dropped out of the study (*χ*2 (1, *N* = 33) = 22.63, *p* < 0.000).

### Outcome assessment

The primary and secondary outcome measures are summarised in Table 1. Preliminary comparative three time-point analyses indicated there was a significant increase in reported use of an additional lubricant during condom use with a sexual partner between baseline (T1) and T3 (*p* = 0.008). There was also an observed decline in the mean scores of the condom use attitudes scale and condom use experiences scale over the intervention period. Furthermore, paired *t* tests showed a decline in the number of reported condom use errors and problems at last use between T1 and T2, (*t*(13) = 2.78; *p* = 0.016, 95% CI [0.29–2.28]), and improvements in participants’ motivation to use condoms between T2 and T3, (*t*(20) = 2.09; *p* = 0.049, 95% CI [0.00–0.67]).

**Table 1:** Three-time point (T1, T2, T3) comparison of selected outcome measures

Outcome measures	T1	T2	T3	Comparative analysis
Condom use at last intercourse, %	57.9	72.2	53.3	^a^Wald *χ*2 = 0.46, *df* = 2, *p* = 0.101
Consistent condom use in the last 4 weeks, %	52.6	50.0	46.7	^a^Wald *χ*2 = 0.15, *df* = 2, *p* = 0.927
Lubricant use in last 4 weeks, %	13.3	53.3	63.6	^a^Wald *χ*2 = 9.71, *df* = 2, *p* = 0.008
Condom use attitudes, M	4.15	4.06	3.90	^c^*F*(1.35,25.72) = 1.02, *p* = 0.347
Condom use errors and problems, M	2.13	0.88	1.25	^b^*F*(2,14) = 1.91, *p* = 0.184
Condom use fit and feel, M	8.71	7.71	8.57	^b^*F*(2,12) = 1.16, *p* = 0.347
Condom use experiences, M	3.37	3.08	2.82	^c^*F*(1.05,6.31) = 3.282, *p* = 0.117
Condom use self-efficacy scale, M	3.91	3.92	4.03	^c^*F*(1.25,23.67) = 0.432, *p* = 0.560
Condom use motivation scale, M	4.14	4.00	4.33	^b^*F*(2,40) = 2.662, *p* = 0.082

^a^Generalised estimating equation: T3 as the reference category

^b^ANOVA with repeated measures

^c^ANOVA with repeated measures with a Greenhouse-Geisser correction

### Process evaluation interviews

The participants interviewed were overwhelmingly supportive of the intervention and would recommend the programme to their friends. Many of the young men reported that they were previously unaware of the variety of condoms available to them and were thankful of the opportunity to try out new brands. Despite initial reservations regarding workload and resource implications of being involved in the HIS-UK study, the health professionals were very positive about the intervention and engagement was good. In one recruitment centre, changes in condom distribution had already occurred as a result of being involved in HIS-UK. Recruitment was facilitated by the use of a variety of high-quality advertising materials and the use of an online registration website, and distribution of the kit by the study researcher meant that health care staff involvement time was kept to a minimum.

The interviews did reveal areas where participants and health professionals thought improvements could be made. For example, it was recommended that a visual tracking system was included on the questionnaires so participants could see how they were progressing on the survey tool. An option to provide further details to questions (open text boxes) was also desired, and a reduction in the number of (perceived repetitive) questionnaire items was requested. Furthermore, the young men felt that the intervention would benefit from the inclusion of strategies to reduce the impact of condom application (i.e. ‘breaking the mood’). Greater emphasis in the literature provided (participant information sheet, instruction guide, website etc.) on the need for, and advantages of, self-practice was another recommendation made. The health professionals felt that engagement of black and minority ethnic (BME) groups could be strengthened and that religious views regarding masturbation may prevent some young men from participating. A summary of all the ‘lessons learned’ following participant and health professional feedback in the process interviews is presented in Table 2.

**Table 2:** Summary of the lessons learned from the follow-up participant interviews

Topic	Participants	Health professionals
Context		Difficult working with youth workers who are already stretched; Timing to start recruitment (e.g. exams for students) was difficult; Funding cuts affected condom availability in community settings
Fidelity	Make the solo-condom testing clearer in all written and verbal communication	
Reach	Targeted appropriate populations, but shy/embarrassed people may be hard to reach	BME groups hard to reach; religious views may be a barrier
Recruitment	Make contact during long gaps in between study activity; reminders were good	Adverts, cards, posters and website were well-received Researchers’ ability to join in with youth organised activities is important
Credible source	Website/university information important, as well as researchers’ background	Researcher came across as knowledgeable about sexual health issues and approachable
Condom demonstration	Several participants who thought they were skilled at application still got things wrong and found demo useful	Important to include a ‘reminder’ and to ensure competency
Rating form and questionnaires	Some said these were too long. Would be good to have open-text questions on rating forms and have a visual tracking system to see progress on the questionnaires	
Impact of study	Most had no awareness about variety of condoms and lubes available; identified condoms they liked and felt more confident with	Initial worry about additional workload (a bit of resistance); make clearer realistic expectations on workload. Has already changed practice—services are offering a wider variety of condoms
Anything we missed? Possible improvements	Transgender and non-binary people—maybe our information does not make clear enough that HIS-UK is suitable for all. The ‘interruption’ condoms are to sex—need ideas on how to reduce	Be opportunistic—interview immediately rather than appointments if possible

## Discussion

The results of the feasibility study and feedback gathered during the evaluative interviews provide useful insights on how successful we have been in adapting the US developed KIHIS for use in the UK. The feasibility study highlighted that our recruitment strategy was appropriate and we achieved our target sample size within the defined time-frame. However, a face-to-face recruitment approach in community and educational settings was deemed somewhat resource-intensive and consideration should be given to alternative recruitment options such as the utilisation of targeted social media advertisements and the use of peer-promoters. Moreover, young men from Black and minority ethnic communities were under-represented in the sample and future studies might employ more focused recruitment activities. In the consultation phase of the study, the potential impact of cultural taboos regarding masturbation and self-pleasure on participation was discussed, but we did not test any strategies to try to assess or reduce this as a potential barrier to recruitment.

Participants were able to self-register for the study, and it was unfortunate that several men who expressed an interest in participating were unable to find an appropriate time to meet with the RA to receive their condom kit and/or did not show up to appointments. Furthermore, one of the initial screening questions (*Do you sometimes go without condoms?*) potentially allowed young men who were already reasonably competent users of condoms to participate in the programme. A small alteration to the wording, such as the non-use of condoms with new or casual partners, would ensure those at greatest risk of STIs are targeted more directly.

The use of a web-based data collection interface was considered highly acceptable to the target group of young men. Furthermore, text messaging and e-mail proved to be suitable methods of communication and, along with relatively modest incentives, ensured 64% retention at 8-week follow-up (T2) and100% retention between T2 and T3. Despite comparing favourably to other similar studies, this fell just short of our minimum target retention rate [32, 33]. During the qualitative interviews, participants recommended that researchers maintain more regular communication with participants during their involvement to increase the likelihood of retention. In addition, we propose supplying participants with regular supplies of their preferred condom type and lubricant as a way to further incentivise.

The feasibility study established that the outcome measures tested were fit for purpose with good inter-item coefficient scores. However, the Condom Use Fit-and-Feel Scale could be extended (and potentially improved) by exploring the inclusion of further relevant items drawn from the other scale items. Furthermore, the current measures of condom use pleasure and sensation were limited to the condom rating forms and could be enhanced by additional inclusion in the T1–T3 surveys.

There were some promising signs from the analyses of the T1–T3 data collected that the HIS-UK intervention may have a positive impact on variables known to be associated with effective and consistent condom use (including condom use attitudes, use experiences, motivation and use of additional lubricant), as has been shown in the testing of KIHIS [15, 16]. These, however, need to be assessed within a fully powered trial before any definitive conclusions can be drawn.

Participant feedback indicated strong acceptance and approval for the intervention and the directed self-practice tasks (86% of young men completing the study were compliant in testing eight different condoms). Indeed, many participants reported that prior to participating in the study they had not given much thought to testing out different condoms for fit and feel, nor to self-practice of condoms in the absence of a sexual partner. That said, when rates of condom rating compliance were examined it was clear that all participant drop-out occurred soon after the condom kits were received—during the initial 2-week condom testing period. Feedback during the follow-up interviews highlighted the need to make all recruitment advertising more explicit regarding the role of solo-testing in the study to ensure that all potential participants are fully aware of what the study involves when they register.

The HIS-UK intervention is novel in that it focuses on improving condom use experience, in particular the fit and feel of condoms during use. A recent review of the literature suggested that only a few previous interventions have had this focus (Anstee S, Graham CA, Stone N, Shepard J, Brown K, Newby K, Ingham R. The evidence for behavioural interventions addressing condom use fit and feel issues to improve condom use: A systematic review. Submitted). Our intervention is also brief and home-based and, thus, should be cost-effective and easy to implement in sexual health clinics as well as community settings. Indeed, NICE guidance recommends providing a range of condom types and distribution schemes to meet the differing needs of young people [13].

## Conclusion

Based on our evaluation of the HIS-UK feasibility study, we conclude that the intervention is acceptable to young men and health promotion professionals and the research design, evaluative tools and outcome measures are appropriate. However, some further development and subsequent piloting of HIS-UK is required prior to testing the intervention in a large-scale randomised controlled trial. Acceptance to randomisation would need to be tested, and future studies might also evaluate the use of a video presentation of the study introduction and condom demonstration, as compared to face-to-face delivery (as in this study). This would provide consistency and standardisation in delivery and could reduce recruitment time and implementation costs, and help retain participants lost following registration. Outcome measurement in future trials should also include STI biomarkers to test the effectiveness of HIS-UK for reducing STI incidence.

## Abbreviations

ANOVA: Analysis of variance
HIS-UK: Home-based intervention strategy UK
KIHIS: The Kinsey Institute Homework Intervention Strategy®
NICE: National Institute of Health and Care Excellence
RA: Research assistant
STI/STIs: Sexually transmitted infection/s
UK: United Kingdom
US: United States
